# c-MYC inhibition impairs hypoxia response in glioblastoma multiforme

**DOI:** 10.18632/oncotarget.8921

**Published:** 2016-04-22

**Authors:** Maria Patrizia Mongiardi, Mauro Savino, Maria Laura Falchetti, Barbara Illi, Francesca Bozzo, Cristiana Valle, Manuela Helmer-Citterich, Fabrizio Ferrè, Sergio Nasi, Andrea Levi

**Affiliations:** ^1^ Institute of Cell Biology and Neurobiology, CNR, c/o CERC, 00143 Rome, Italy; ^2^ Nucleic Acids Laboratory, Institute of Molecular Biology and Pathology, National Research Council (IBPM-CNR) and Department of Biology and Biotechnologies, Sapienza University, 00185 Rome, Italy; ^3^ Department of Biology, University of Rome Tor Vergata, 00133 Rome, Italy; ^4^ Fondazione Santa Lucia IRCCS, c/o CERC, 00143 Rome, Italy; ^5^ Centre for Molecular Bioinformatics, Department of Biology, University of Rome Tor Vergata, 00133 Rome, Italy; ^6^ Department of Pharmacy and Biotechnology (FaBiT), University of Bologna Alma Mater, 40126 Bologna, Italy

**Keywords:** HIF, c-MYC, hypoxia, glycolysis, glioblastoma

## Abstract

The c-MYC oncoprotein is a DNA binding transcription factor that enhances the expression of many active genes. c-MYC transcriptional signatures vary according to the transcriptional program defined in each cell type during differentiation. Little is known on the involvement of c-MYC in regulation of gene expression programs that are induced by extracellular cues such as a changing microenvironment. Here we demonstrate that inhibition of c-MYC in glioblastoma multiforme cells blunts hypoxia-dependent glycolytic reprogramming and mitochondria fragmentation in hypoxia. This happens because c-MYC inhibition alters the cell transcriptional response to hypoxia and finely tunes the expression of a subset of Hypoxia Inducible Factor 1-regulated genes. We also show that genes whose expression in hypoxia is affected by c-MYC inhibition are able to distinguish the Proneural subtype of glioblastoma multiforme, thus potentially providing a molecular signature for this class of tumors that are the least tractable among glioblastomas.

## INTRODUCTION

Many solid tumors reside in a hypoxic environment when their mass exceeds the oxygen diffusion limit. Lack of oxygen reprograms gene expression to favor cellular adaptation to reduced oxygen concentration by decreasing mitochondrial respiration and promoting glycolysis as a main source of ATP production [1]. Importantly, the shift to glycolysis contributes to cancer cell proliferation because many glycolytic intermediates are substrates for anabolic pathways crucial for the synthesis of macromolecules and organelles [2]. Possibly because of these growth promoting effects, many cancers exhibit a glycolytic phenotype even at normal level of oxygen, a phenomenon known as aerobic glycolysis or Warburg effect [3] [4]. Hypoxic reprogramming of gene expression is to a large extent mediated by Hypoxia Inducible Factors HIF-1 and HIF-2, heterodimeric transcription factors made up of the common, constitutively expressed subunit, ARNT/HIFB, and the oxygen regulated subunits HIF1A and HIF2A respectively. The quantities of HIF1A and HIF2A are mainly controlled through oxygen-dependent protein turnover. There is wide evidence of a complex crosstalk between HIFs and c-MYC, a transcription factor deregulated in most human cancers [5]. Transcriptional activation by c-MYC requires hetero-dimerization with MAX and binding to consensus DNA recognition elements, the E-boxes. HIFs and c-MYC were shown to control both converging and opposing intracellular pathways [6]. HIF-1 and a deregulated c-MYC in cancer cells cooperatively induce transcription of genes involved in hypoxic adaptation such as genes regulating metabolic reprogramming and angiogenesis [7]. HIF-1 was also shown to directly inhibit c-MYC transcriptional activity at some c-MYC target genes, by influencing c-MYC interactions with protein partners and by DNA-binding site competition [8]. HIF-1 and c-MYC differently regulate mitochondria mass, the first promoting mitophagy [9] the second inducing mitochondria biogenesis [10]. Glioblastoma Multiforme, GBM, the most aggressive brain tumor, is characterized by large necrotic/hypoxic areas and extensive angiogenesis. c-MYC activity is frequently altered in GBMs [11] and, like hypoxia [12], is associated with maintenance of GBM cancer stem cells [13]. Therefore GBM represents a convenient paradigm to investigate the reciprocal influence of HIFs and c-MYC in tumor growth and progression. To interfere with c-MYC activity we used the dominant negative c-MYC inhibitor, Omomyc, an engineered miniprotein consisting of the bHLHZip domain of c-MYC with four specific point mutations that allow its homodimerization as well as heterodimerization with both MAX and c-MYC [14]. Omomyc antagonizes the transcriptional transactivation activity of c-MYC by preventing c-MYC binding to E-boxes and affecting the c-MYC interactome. Here we investigated the impact of Omomyc-mediated c-MYC inhibition on the HIF-1-dependent transcriptional response of GBM cells. Omomyc altered the induction of a subset of HIF-1 target genes, the majority of them being blunted in their hypoxia-dependent activation. This was accompanied by a decreased glycolysis and increased oxidative phosphorylation. Based on HIF-1 targets modulated by Omomyc in hypoxia, we established a signature that identifies the most therapy-resistant Proneural GBM subtype [15].

## RESULTS

### MYC inhibition alters the transcriptional response to hypoxia in GBM cells

**Figure 1:**
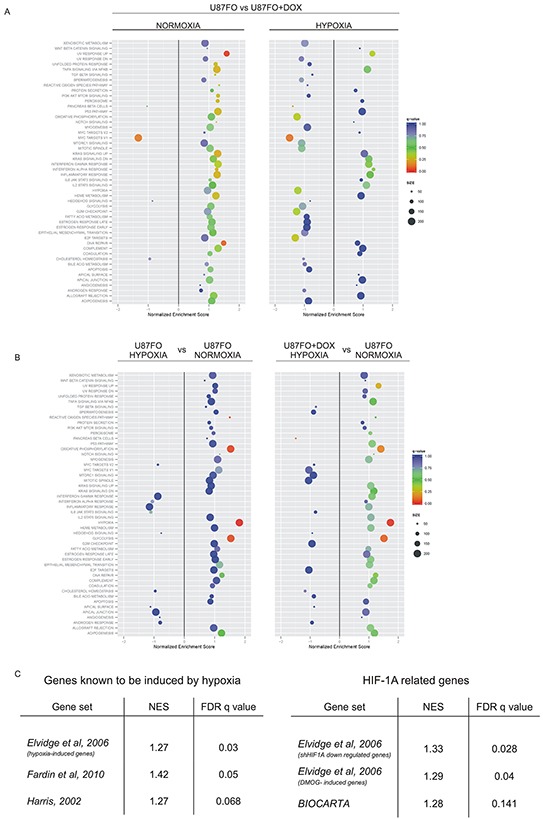
Hypoxia and Omomyc cross-regulate gene expression program **A.** GSEA analysis of Omomyc regulated genes in normoxia (left panel) and in hypoxia (right panel) for all the hallmark gene sets of the Molecular Signature Database, MSigDB. **B.** GSEA analysis of hypoxia regulated genes in U87FO cells. Hypoxic versus normoxic U87FO cells (left panel) and hypoxic, DOX-treated, U87FO cells versus nomoxic U87FO cells (right panel). **C.** GSEA based on the comparison between hypoxic U87FO cells versus hypoxic, DOX-treated U87FO cells. The analyses were performed on RNA-seq data from 3 biological replicates.

To explore the possible role of c-MYC in tuning gene expression changes in hypoxia, we transduced the glioblastoma cell line U87MG with a lentiviral vector encoding a doxycycline (DOX)-inducible, Flag-tagged Omomyc construct. Upon hygromycin selection, the surviving population was named U87FO. Control cells were obtained by infection with lentiviral particles encoding a DOX-inducible GFP (U87GFP). U87FO cells exhibited a rapid and strong expression of Omomyc after DOX treatment, with a peak of expression between 24 to 48 hours (not shown). U87FO cells were grown for 48 hours in the absence or presence of DOX and exposed to moderate hypoxia (2% O_2_) or kept in normal oxygen concentration (normoxia) for an additional 5 hours. Total RNA extracted from the cells was subjected to deep sequencing. We employed gene set enrichment analysis, GSEA [16] [17] to establish which are the main cellular processes altered in U87FO cells upon c-MYC inhibition and exposure to hypoxia. We evaluated the enrichment of the 50 gene sets, defined as Hallmark gene sets [18], in the Molecular Signature Database, MSigDB, of the Broad Institute, (http://www.broadinstitute.org/gsea/msigdb/index.jsp). We first assessed how the transcriptional response to c-MYC inhibition is influenced by hypoxia (Figure 1A). DOX treatment in normoxia significantly downregulated the c-MYC target gene set (Normalized Enrichment Score NES -1.32, False Discovery Rate FDR q- value ≤ 0.05) and upregulated the DNA damage gene set and the one of transcripts up regulated by UV (NES, 1.48 and 1.58 respectively, FDR q- value ≤ 0.05), Figure 1A. Such a transcriptional response to c-MYC inhibition was largely perturbed in hypoxia (Figure 1A right panel). The c-MYC target gene set remained significantly downregulated by Omomyc, whereas the UV response UP and DNA repair gene sets lost their strong correlation with Omomyc expression. This indicated that hypoxia altered the Omomyc-dependent changes in expression of many genes present in these gene sets. We next investigated how the transcriptional response of U87FO cells to hypoxia is influenced by c-MYC inhibition. Exposure to hypoxia in the absence of DOX led, as expected, to a significant enrichment of gene sets commonly related to hypoxia such as glycolysis, reactive oxygen species (ROS) and oxidative phosphorylation (Figure 1B, left panel). In DOX-treated hypoxic cells, the enrichment of ROS related gene set lost significance, as shown by the strong q-value increase. More subtle effects were observed in the hypoxia, glycolysis and oxidative phosphorylation gene sets. To directly asses how c-MYC inhibition altered their expression, we performed GSEA of hypoxic U87FO cells with respect to hypoxic, DOX-treated, U87FO cells, for all gene sets of the Broad Institute data bank identified by keywords such as, hypoxia, oxidative phosphorylation and glycolysis. As shown in Figure 1C gene sets identified upon exposure to hypoxia [19] [20], hypoxia-mimetic drugs, upregulation of HIF1A or its depletion by RNA interference [21] were significantly enriched in hypoxic U87FO cells. Gene expression profile of selected sets related to glycolysis, angiogenesis and hypoxia responsiveness are depicted in Supplementary Figure S1. Gene sets identified by HIF-2 as a keyword were not enriched, possibly because HIF2A protein level does not substantially change in U87FO cells upon 5 hours of hypoxia, not shown. We thus decided to limit our analysis on those transcriptional responses to hypoxia which are mediated by the activation of HIF-1.

### Omomyc reduces HIF1A binding to the promoter of specific target genes

**Figure 2:**
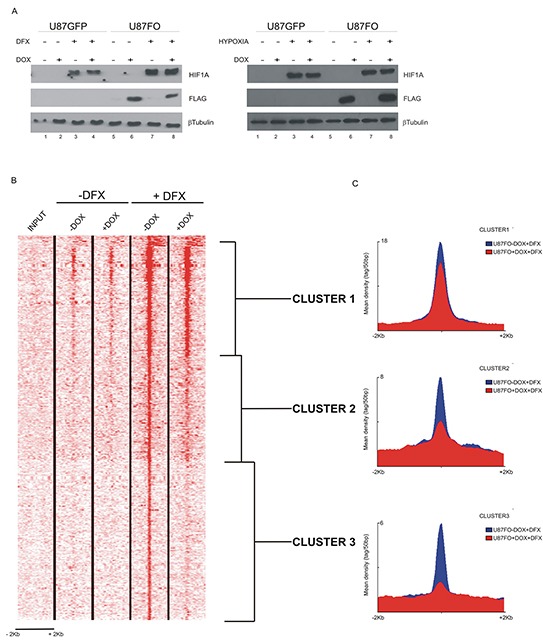
c-MYC inhibition destabilizes HIF1A binding to target promoters **A.** Western blot analysis on U87GFP and U87FO cells pretreated for 48h with DOX and then exposed for 5h to DFX (left panels) or to hypoxia (right panels). DOX treatment induced Omomyc expression (Lanes 6 and 8). DFX- and hypoxia -induced HIF1A protein stabilization is not impaired by Omomyc expression (Lanes 3, 4, 7 and 8). **B.** ChIP-seq analysis of HIF1A binding to DNA of U87FO cells without or with Omomyc induction for 48 hours and without or with DFX treatment for 5h. Shown are ± 2kb regions centered on all the TSSs and the color scale represents tags per 50bp. **C.** Density maps of the three HIF1A gene clusters determined by Seqminer.

U87FO cells were grown for 48 hours with or without DOX and exposed for an additional 5 hours to vehicle or to 130 nM deferoxamine (DFX), a hypoxic mimetic drug that prevents the rapid oxygen-dependent degradation of HIF1A. Omomyc *per se* did not alter HIF1A protein level with or without DFX treatment (Figure 2A left). Similar results were observed in response to hypoxia, Figure 2A right. By Chromatin immunoprecipitation (ChIP)-sequencing analysis using a HIF1A antibody, we found that HIF1A bound to approximatively 1200 promoters, being strongly enriched in the chromatin region near the transcription starting site (Figure 2B). DOX treatment caused a reduction of HIF1A binding to promoters, to a variable extent. Importantly we previously showed that Omomyc does not bind to HIF-1A [22]. We identified three gene clusters: i) cluster 1, where HIF1A binding was strong and minimally inhibited by Omomyc, ii) cluster 2, characterized by a more modest HIF1A binding and a more pronounced Omomyc inhibitory effect, and iii) cluster 3 with a weak HIF1A binding, essentially limited to the transcription start site, strongly inhibited by Omomyc (Figure 2C).

### Omomyc alters the hypoxic expression of a subset of HIF-1 target genes in U87FO cells

**Figure 3:**
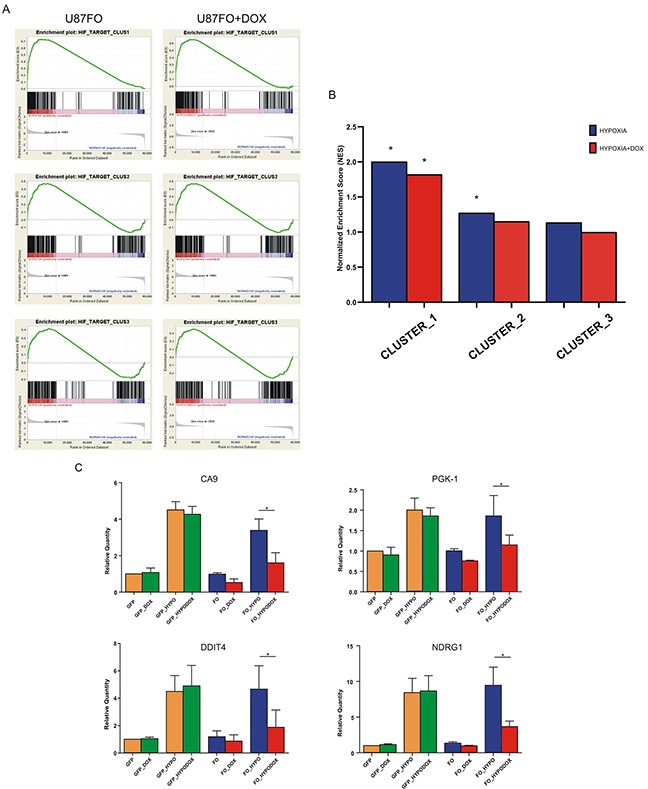
c-MYC regulates HIF1A transcription activity **A.** GSEA of the three HIF1A gene clusters. The analysis were performed on gene expression data obtained from hypoxic versus normoxic U87FO cells (left) and from hypoxic-DOX-treated U87FO cells versus normoxic U87FO cells (right). **B.** Graph bars indicate NESs * FDR q value < 0.05. **C.** Quantitative Real Time RT-PCR on CA9, PGK1, DDIT4 and NDRG1 in U87GFP cells and U87FO cells. Relative Quantities were calculated normalizing for TBP and are given relative to U87GFP. n= 3 biological replicates * p value < 0.05.

To assess the hypoxia-dependent regulation of HIF1A-bound genes and the consequence of c-MYC inhibition, we evaluated the enrichment of each cluster by GSEA. The three clusters showed different enrichment scores that reflected the intensity of HIF1A binding signal (Figure 3A). Indeed cluster 1, with the strongest HIF1A binding, had the best enrichment score (NES 1.99) whereas cluster 3 gene set did not attain a significant enrichment (FDR q value = 0.12), Figure 3B. In accordance to previous results none of the HIF1A bound gene had decreased expression upon hypoxia [23]. Omomyc reduced the enrichment score of all three clusters (Figure 3B) indicating that c-MYC inhibition blunted the transcriptional response of U87FO cells to HIF1A. To identify the HIF1A targets that were more significantly affected by Omomyc, we used the RNA-seq data to compare - in cells previously treated or not with DOX - the expression change in hypoxia of each HIF1A bound gene. Table 1 shows that 85 genes were significantly less induced in hypoxia upon Omomyc expression (Omo-down genes) and 25 genes were more induced (Omo-up genes). Less than 10% of the Omo-down genes (9 out of 85) - were downregulated by DOX in normoxia (Table 1, in italic and underlined). Therefore c-MYC inhibition appears to selectively impair the transcriptional enhancement by hypoxia of Omo-down genes rather than their basal expression. Similarly, Omomyc preferentially increased transcription of Omo-up genes in response to hypoxia, since only about a quarter of them were upregulated in normoxia as well. Real time RT-PCR on selected Omo-down genes strongly induced by HIF1A in hypoxia, Carbonic Anhydrase-9 (CA9), Phosphoglycerate Kinase-1 (PGK1), DNA-damage Inducible Transcript-4 (DDIT4) and N-MYC Down Regulated Gene-1 (NDRG1), was used to validate the RNA-seq data, Figure 3C. Moreover in U373FO cells, a second GBM cell line infected with pSLIK-FO (Supplementary Figure S2a), the expression of three of those genes, CA9, DDIT4, NDRG1, was similarly modulated by Omomyc, whereas PGK1 could not be compared because not responsive to hypoxia in U373FO cells (Supplementary Figure S2b). DOX treatment also blunted the induction of CA9, DDIT4 and PGK1 upon treatment with DFX (not shown) and in a U87MG-derived cell line harboring a mutant HIF1A resistant to oxygen-dependent degradation (Supplementary Figure S3a and S3c). Similarly to Omomyc expression, c-MYC inhibition by RNA interference reduced hypoxia-dependent transcription of CA9, DDIT4, PGK1 genes, Supplementary Figures S4a and S4b. This suggests that Omomyc impairment of the hypoxic induction of gene expression reflects c-MYC inhibition rather than off target effects.

**Table 1:** Omo-down and Omo-up genes list

OMO-DOWN		OMO-UP
Number of genes: 85		Number of genes: 25
ADAT2	KIF11	ANKRD37
*ADCY8*	KNTC1	ATP6V0E1
ADD3	KPTN	C15orf39
ALDH1A3	LTBP4	CIB2
ARID2	*LY6K*	*DHRS13*
*ARL6*	MME	DHX58
ASPH	MT1X	DYNLL1
ATXN1	MT2A	EFEMP2
BNIP3	NAMPT	FAM131C
BRCA2	NDRG1	FLOT2
C10orf10	NEDD4L	*GPRC5A*
C1QL1	NGLY1	*GSG1*
C8orf58	NR2F1	MAP1S
*CA9*	NR2F2	MIR155HG
CBX5	NTSR1	NUDCD1
CCDC107	PDK1	P4HTM
CHD1	PFKFB4	*PDZD7*
CRYBG3	PGK1	*PPM1J*
CSTB	PLOD2	PRICKLE3
CTDSP1	POLR2H	RNASE4
CUL4A	*POU2F1*	*SERTAD1*
DARS	PUS7L	SH3BGRL3
*DDIT4*	RARA	SHISA4
DDX18	RELL2	SURF4
DDX50	RLF	*UBC*
DHX15	RPS12	
E2F8	RPS13	
ERO1L	SAP30	
EXOC5	SGOL2	
FAM117B	SIGMAR1	
FAM13A	SMARCAD1	
FAM162A	SMC2	
GBE1	SMS	
GLRX	SNTB1	
GTF2E1	*SRD5A3*	
HMOX1	*TET2*	
HSP90B1	TIA1	
IGFBP1	TMEM158	
IMMP2L	*TRIM9*	
JUN	TRMT61A	
KDM3A	UCN2	
	YTHDF2	
	ZNF292	
	ZNF789	

HIF1A target genes which induction under hypoxia resulted inhibited (Omo-down) or amplified (Omo-up) by Omomyc expression. Altered induction in hypoxia by Omomyc was calculated with a paired T-test on the log2 of fold change of hypoxic induction without or with DOX for each of the all three biological replicates of the RNA-seq. Genes in italic and underlined are those altered by DOX treatment also in normoxia.

### Omomyc expression increases mitochondrial functionality in hypoxic cells

**Figure 4:**
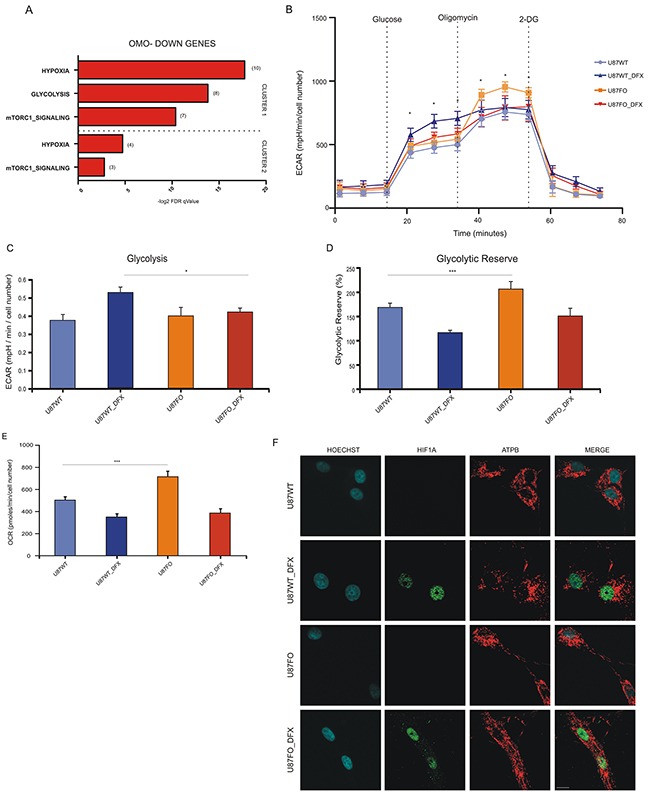
HIF1A controls metabolic profile in a c-MYC dependent manner **A.** Computational overlap analysis between Omo-down genes and Hallmark gene sets from MSigDB. **B.** Extracellular acidification rate (ECAR) in U87WT and U87FO treated or not with DFX. Glucose, Oligomycin A and 2-Deoxy-D-glucose (2-DG) were injected at the indicated time points. Data show mean ± SD. * p<0.05. See also Supplementary Figure S5a. All cellular groups employed were treated with DOX in order to avoid out of target effects due to this treatment. **C.** Glycolysis in U87WT and U87FO treated or not with DFX. Glycolysis is calculated by subtracting basal ECAR levels to ECAR levels measured after glucose injection (see Supplementary Figure S5a). Bar graphs represent mean ± SD of the three measurements in basal and after glucose injection at the indicated time points. * p < 0.05. **D.** Glycolytic reserve in U87WT and U87FO cells treated or not with DFX. Glycolytic reserve is calculated by subtracting ECAR measured after glucose injection from ECAR measured after oligomycin A injection (see Supplementary Figure S5a). Bar graphs represent mean ± SD of the three measurements after glucose and oligomycin A injection at the indicated time points. * p < 0.05. **E.** Oxygen consumption rate (OCR) in U87WT and U87FO cells treated or not with DFX. Data presented as mean ± SD of the three measurements in basal conditions. *** p< 0.005. **F.** Staining of ATPB and HIF1A in U87WT and U87FO cells treated or not with DFX. Scale bar, 10μm. All cellular groups employed were treated with DOX in order to avoid out of target effects due to this treatment.

By GSEA analysis, Omo-down genes belonging to clusters 1 and 2 showed high correlation with hypoxia-regulated pathways and had significant enrichments for two pathways associated with cellular metabolism: glycolysis and mTORC1 signaling, Figure 4A, suggesting that the energetic metabolism of U87MG cells in hypoxic conditions could be altered by c-MYC inhibition. No overlap was found for Omo-down genes in cluster 3 neither for the Omo-up genes. We then used the SeaHorse Bioscience XF Glycolysis Stress Test (http://www.seahorsebio.com) to measure the energetic metabolism of Omomyc-expressing and control cells. Since DOX may affect mitochondria function [24] we compared the metabolic profile of U87MG wild type (U87WT) and U87FO cells, both treated with DOX. Cells were grown with DOX for 32 hours followed by 16 additional hours with or without DFX. U87WT cells displayed a typical Extra-Cellular Acidification Rate (ECAR), with increased acidification upon glucose injection and an appreciable glycolytic reserve measured upon poisoning mitochondria with oligomycin A, Supplementary Figure S5a. Upon DFX treatment, U87FO cells displayed a reduced glucose-stimulated ECAR increase with respect to U87WT cells, Figure 4B and 4C, and a higher glycolytic reserve with or without DFX treatment Figure 4D. Accordingly, Omomyc-expressing cells had an oxygen consumption rate, OCR, higher than wild type cells, Figure 4E. Similar results were obtained in cells treated with dimethyloxalylglycine, DMOG, a different inhibitor of HIF1A degradation, not shown. Next we stained U87WT and U87FO cells with antibodies against the mitochondrial protein ATP synthase subunit β, ATPB, to evaluate whether mitochondrial morphology was altered by Omomyc expression. In normoxia, both U87WT and U87FO cells contained healthy fused mitochondria Figure 4F. After DFX treatment or in hypoxic condition a clear mitochondrial fragmentation phenotype was noted in U87WT cells, Figures 4F and Supplementary Figure S5b. Omomyc expression inhibited mitochondrial fragmentation and restored an overall normal mitochondrial morphology in most cells Figures 4F and Supplementary Figure S5b. All these data suggest that c-MYC inhibition drives important metabolic changes in glioblastoma, so that the cells better preserve functional mitochondria in hypoxia and have a more pronounced oxidative phosphorylation phenotype and a reduced glycolytic one.

### Omo-down and Omo-up signatures are predictive of GBM subtypes

**Figure 5:**
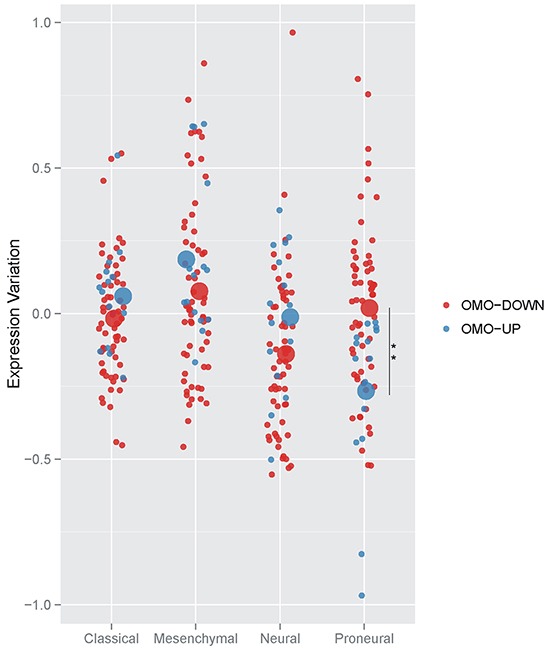
Averaged expression variation of Omo-down and Omo-up genes in GBM subtypes Side-by-side comparison of the expression variation distributions for Omo-down and Omo-up gene sets. For each GBM sub-type, the average expression variation was computed for each gene in the gene sets (small dots), and then represented as the mean value for all genes (big dots). For each possible pair of GBM subtypes, the statistical significance of differences in the average value distributions was estimated by a T-test. For each GBM subtype, the statistical significance of differences in the average value distributions between Omo-down and Omo-up lists was estimated by a T-test. ** p < 0.01.

We sought to test whether the expression of Omo-down and Omo-up genes associates with features and treatment response of GBM patients. Meta-analysis of gene expression levels in 202 patients led to a robust classification of GBM into four subtypes (Proneural, Neural, Classical and Mesenchymal) [15] which stratify the patients for their genomic abnormalities and response to therapy. To assess whether the Omo-down and Omo-up genes show similar expression trends in the four subtypes, we retrieved the normalized expression variation data from the Cancer Genome Atlas, and computed the average expression of Omo-down and Omo-up genes in the four GBM types, Figure 5. We were able to unambiguously map 61 genes out of 85 in the Omo-down list, and 13 out of 25 in the Omo-up list to the array-based TCGA GBM classification data. The Omo-down genes show a broad range of expression variation, even if their average expression is similar in each GBM subtype. On the other hand, the Omo-up genes expression seems to differ more substantially in the different GBMs. By comparing the expression variation levels of Omo-down and Omo-up genes in the GBM subtypes we observed a strong significant difference in and only in the Proneural subtype, Figure 5. Testing sets of the same size of the Omo-up and Omo-down sets generated by sampling for 10,000 times from the complete HIF1A target genes only in one case out of ten thousand, the sampled gene set showed a lower average expression than the Omo-up genes, Supplementary Table S1. Presently it is not clear which are the biological basis, if any, for this correlation between the Proneural subtype and the sets we identified by perturbing the hypoxic response of U87MG cells. We however believe that the difference between Omo-up and Omo-down expression may be used as an additional signature of Proneural GBMs.

## DISCUSSION

c-MYC deregulation is involved in most human cancers and hypoxia is a main contributor to drug resistance in cancer through different mechanisms. In GBM, hypoxia reduces the response to temozolomide, a DNA damaging molecule constituting the standard drug treatment for GBM, by inhibiting apoptosis [25]. We found that, in U87MG cells, the c-MYC inhibitor Omomyc significantly affected the expression of a set of genes involved in DNA repair and that five hours of hypoxia were sufficient to appreciably alter the cellular transcriptional response to c-MYC inhibition, compare Figure 1A right and left panels. Exposure to hypoxia of U87FO cells resulted in a significantly enriched expression of gene sets related to hypoxia, glycolysis, oxidative phosphorylation and ROS (Figure 1B left panel), which was minimally altered by Omomyc, as shown by the small changes in FDR q-value and NES upon DOX treatment (Figure 1B left and right panel). Instead, Omomyc strongly affected the expression of the set of genes directly regulated by the transcription factor HIF1A, a main mediator of the cell response to hypoxia, Figure 1C. Omomyc reduced HIF1A binding to target promoters and changed HIF1A-dependent induction of selected target genes. The amount of gene expression change caused by Omomyc was not correlated to the decrease of HIF1A binding to the gene promoters, suggesting that different mechanisms account for c-MYC-dependent regulation of HIF1A binding to promoters and transcriptional activation. It is well established that HIF1A binds to easily accessible regions of DNA marked by specific histone modification [26] and that c-MYC promotes chromatin opening [27] [22] [28]. Possibly Omomyc reduces access of HIF1A to those promoters which are kept in an open configuration by c-MYC-dependent histone modifications. Many genes in cluster 1 display a measurable HIF1A binding even prior to DFX treatment (lanes –DFX in Figure 2B). In these conditions the amount of HIF1A protein is quite low and therefore it binds only to high affinity sites. Conceivably HIF1A bound to these sites maintains an accessible chromatin by recruiting histone modifiers such as p300/CBP [29] thus relieving the necessity for an active c-MYC for additional HIF1A binding upon DFX addition. This may account for unimpaired HIF1A binding to cluster 1 genes upon Omomyc expression. Considering both Omo-up and Omo-down genes, c-MYC inhibition alters the expression of about 10% of HIF1A targets. Since our analysis was limited to a precise time point, 5 hours, this is likely an underestimation of the number of genes requiring c-MYC for optimal induction by HIF-1. Many mechanisms may account for c-MYC fine tuning the expression of HIF1A-induced genes: c-MYC binding in *cis* may be required for recruitment of transcriptional activators to the promoter of Omo-down genes and for recruitment of transcriptional repressor to the promoter of Omo-up genes. Otherwise c-MYC may act in *trans* possibly by regulating the level of transcription factors which cooperate with HIF1A or microRNAs that affect the stability of HIF1A regulated mRNAs. Recently it was shown that a CDK8-Mediator complex increases the expression in hypoxia of many, but not all, HIF1A target genes by alleviating RNA polymerase II pausing [30]. It is known that c-MYC also promotes transcriptional pause release [31] and that through this mechanism it amplifies the expression of many genes in cancer [32] as well as in normal cells [33]. It is possible that c-MYC-dependent release of pausing polymerase is required for optimal hypoxic expression of Omo-down genes. We found that of Omo-down genes set is significantly enriched for genes involved in regulation of glycolysis. Importantly direct measurements showed that Omomyc reduced the shift to glycolysis in response to DFX. We also observed that mitochondrial morphology, a proxy of mitochondrial function, is better preserved by Omomyc expression both in hypoxia and in response to hypoxic mimetic drugs. These findings are reminiscent of the Warburg effect; however we observed them only in hypoxia or hypoxia-mimetic conditions. Tumor switching from mitochondrial respiration to glycolysis is accompanied by mitochondrial hyperpolarization and resistance to apoptosis, conversely, sustained mitochondria respiration promotes ROS increase and cell death in GBM cells [34]. Dichloroacetate, DCA, an inhibitor of pyruvate dehydrogenase kinase, reverts mitochondrial hyperpolarization and induces apoptosis in GBM cells [35]. Interestingly we observed that Omomyc-expressing hypoxic U87FO, are enriched, (FDR q-value = 0.17 NES of - 1.1), for a gene set defined by DCA response in lung cancer and GBM cell lines [36], Supplementary Table S2. Omomyc has anti-cancer effects on a number of different tumors, including GBM [37]. We believe that deregulating HIF1A-dependent cellular adaptation to hypoxia may be part of the therapeutic effect of Omomyc. It is important to identify gene expression signatures which distinguish GBM subtypes and allow to stratify patients according to the more appropriate therapeutic approach. Our analysis shows that high difference between expression variation of Omo-down and Omo-up gene sets strongly characterize Proneural GBM, a subtype particularly resistant to the current standard therapeutic options [15].

## MATERIALS AND METHODS

### Cell cultures and plasmids

Cells were cultured in DMEM (Lonza, Basel Switzerland) with 10% heat-inactivated fetal bovine serum (FBS; Lonza). All cell lines were regularly controlled for mycoplasma contamination. Doxycycline (Sigma; St. Louis MO, USA) was used at a final concentration of 1μg/ml. Cells were subjected to hypoxia using a modular incubator chamber flushed with a gas mixture of 2% O_2_, 5% CO_2_ and 93% N_2_. Deferoxamine (DFX; Sigma) was used at a final concentration of 130 nM. Flag tagged Omomyc and GFP were cloned in pSLIK plasmid [38]. 293T cells were co-transfected with the pSLIK plasmids and pMDL, pGAG and pVSVG plasmids [39] using Lipofectamine (Invitrogen, Carlsbad, CA USA). Forty-eight hours post-transfection retroviral particles were collected and used to infect U87MG cells.

### ChIP-sequencing

Chromatin for ChIP-seq was prepared by fixing U87FO cells in 1% formaldehyde for 10 min, followed by quenching with 125 mM glycine for 5 min. ChIP was performed using anti-HIF1A antibody (NB100-105, Novus Biologicals CO, USA) according to Myers Lab ChIP-seq Protocol (https://www.encodeproject.org/documents/). Crude nuclei, obtained by cells extracted in Farnham lysis buffer, were resuspended in RIPA buffer and sonicated using a Branson Sonifier 250 to shear DNA to an average fragment size of 100-250 bp. Immunoprecipitations were performed using 40 μl of Protein G Dynabeads (Invitrogen) and 5 μg of antibody in each ChIP reaction. After washing and reverse cross-linking, DNA was purified using the QIAquick PCR Purification Kit (Qiagen Inc. Valencia CA, USA). Illumina sequencing libraries were prepared at the Istituto di Genomica Applicata (IGA) facility in Udine and subjected to high-throughput sequencing on the Illumina HiSeq 2500 (www.igatechnology.com/). ChIP-seq reads were analyzed using CAST (Chip-Seq Analysis System Tool), an automated pipeline developed by CINECA and available at https://bioinformatics.cineca.it/. Reads alignment to the human genome assembly hg19 (GRCh37) and peak calling were performed using Bowtie and MACS algorithms within CAST, setting a p-value threshold for peak detection of 1e^−4^. Peaks were annotated to nearest genes by the CAST internal annotator. Processed data were then visualized and subjected to cluster analysis using seqMINER v1.3.3e.

### RNA-sequencing

For RNA-seq, total RNA, from 3 biological replicates, was isolated by Trizol and subjected to DNAse digestion (RQ1 DNAse, Promega WI, USA). DNA-free RNA was purified by PureLink RNA (Life Technologies Carlsbad CA, USA) and 2 μg were used for sequencing analysis. Illumina sequencing libraries were prepared at the IGA and subjected to high-throughput sequencing on the Illumina HiSeq 2000. Read quality was examined using FastQC (v0.11.12 at http://www.bioinformatics.babraham.ac.uk/projects/fastqc/). After adapter sequence removal, read ends were trimmed if base quality scores were lower than Q20 using Trimmomatic (Bolger, Lohse, and Usadel 2014). Reads with the total length shorter than 30 bases or with the fraction of undetermined bases >2% were discarded. The resulting cleaned reads were analyzed with the Tuxedo Suite, comprising of Bowtie (v2.1.0) [40], TopHat (v2.0.9) [41] and Cufflinks (v2.1.1) [42]. We aligned the sequence reads to the reference human hg19 (GRCh37) genome assembly with a tolerance of two mismatches (Bowtie), we identified splicing junctions (TopHat), and we reconstructed transcripts and measured their expression levels (Cufflinks), reported as Fragments Per Kilobase of transcript per Million mapped reads (FPKM). Human gene structures in GTF format were downloaded from Ensembl. Altered induction in hypoxia by Omomyc was calculated with a paired T-test on the log2 of the FPKM fold change after hypoxic induction, without or with doxycycline, for each of the all three biological replicates of the RNA-seq.

### Over-representation GSEA analysis

Gene set enrichment analysis (GSEA) was performed using GSEA software available on Broad Institute website. GSEA software was applied on FPKM expression data. Normalized enrichment scores were considered significantly enriched at FDR q value < 5%, using 1000 permutations of gene sets.

### Reverse transcription and real-time RT-PCR analyses

Two μg of RNA isolated by TRIZOL reagent were retrotranscribed with MLV-Reverse Transcriptase (Promega) according to standard procedures. Fifteen ng of cDNA were used to quantify the transcripts by Real Time RT-PCR using SYBR Select Master Mix (Applied Biosystem Foster City CA, USA) and gene specific-primers, listed in supplemental information. Real-time PCR was performed with the 7900HT Fast Real-Time PCR System by Applied Biosystem. Statistical analysis was performed using Prism software (GraphPad software). Mean values and standard deviation were generated from at least three biological replicates.

### Immunoblotting

Cell extracts were prepared in lysis buffer (50 mM TRIS-HCl (pH 7.5), 250 mM NaCl, 1% NP-40, 5 mM EDTA, 5 mM EGTA, supplemented by protease inhibitors (SIGMA). Thirty μg of protein extracts were separated by sodium dodecyl sulfate-PAGE (SDS-PAGE), blotted onto nitrocellulose membrane and detected with specific antibodies. Immunoblots were revealed by enhanced chemiluminescence (ECL) (Super Signal West Dura, Thermo Scientific Waltham MA, USA). The following antibodies were used: anti-HIF1A (54/HIF1A; BD Transduction Laboratory San Jose CA, USA), anti-FLAG (Flag- M2 Sigma), anti-Myc (c-Myc A190, Bethyl Lab Montgomery TX, USA), anti-βTubulin (DM1A; Sigma).

### Metabolic flux analysis

Seahorse XF96^e^ Analyser (Seahorse Bioscience-Agilent Santa Clara CA, USA) was used to measure ECAR and OCR. Experiments were performed with the XF Glycolysis stress test kit (Seahorse Bioscience) in accordance to manufacturer instructions. Briefly, 10^4^ U87MG and U87FO cells, pre-treated for 32h with DOX, were seeded on poly-l-lysine-coated XF96 microplates in 200 μl DMEM 10% FBS with DOX and treated for additional 16h with or without DFX. Before XF Glycolysis stress assay, medium was replaced with 80 μl glycolysis stress test medium without glucose and cells were incubated in a CO_2_-free incubator at 37°C for 1 hour. XF96^e^ assays consisted of sequential drug injections followed by three cycles of mix, pause and parameters measurements. The first injection consists of a saturating concentration of glucose (10 mM) that stimulates basal glycolytic metabolism, the second contained Oligomycin A (1μM) that inhibits mitochondrial ATP production and shifts the energy production to glycolysis and the last was 2-deoxy-glucose (2-DG, 50 mM), a glucose analogue, which inhibits glycolysis. Data were analysed with Wave software (available on Seahorse Bioscience website) and results are presented normalized to cell number. Statistical analysis was performed using Prism software (GraphPad Prism 6 software). Representative results of a single experiment with n=10 biological replicates are shown. Two independent experiments were consistent.

### Immunofluorescence analysis

Cell cultures were grown on poly-l-lysine-coated glass slides, fixed at room temperature for 10 min with 4% paraformaldehyde in PBS, permeabilized with 0.1% Triton X-100 for 5 min and then washed in PBS. Cells were blocked for 30 min in 2% horse serum in PBS and incubated with primary antibodies (mouse anti-ATPB (Abcam Cambridge, UK) and rabbit anti-HIF1 (Santa Cruz Santa Cruz CA, USA) diluted in blocking solution, for 1 h at 37°C and then with secondary antibodies Cy3- and Alexa488-conjugated (Molecular Probe Eugene OR, USA). After rinsing in PBS, cells were counterstained with 1 μg /ml Hoechst 33342 and examined with a Zeiss LSM 510 Confocal Laser Scanning Microscope. Fluorescence images were processed using ZEN 2009 (Carl Zeiss, Milan, Italy) and CorelDRAW image software.

### Glioblastoma subtypes expression data

Microarray expression data from 202 glioblastoma samples classified into four subtypes [15] were retrieved from the Cancer Genome Atlas (https://tcga-data.nci.nih.gov/docs/publications/gbm_exp). Expression data were measured using three different platforms, scaled and factorized to obtain a single expression estimate for each gene in each sample, and normalized by the median absolute deviation (MAD) to obtain a single variation estimate for each gene [15]. To establish correlations between the GBM phenotypes and the Omo-up and Omo-down gene sets, we computed the average expression variation of the gene sets in the four sub-types, and compared the obtained average expression variation distributions using a T-test. Control gene sets of the same size of the Omo-up and Omo-down sets, were generated by sampling for 10,000 times from the complete HIF1A target genes as detected by ChIP-seq.

## SUPPLEMENTARY METHODS FIGUERS AND TABLES


