# Supplementary material for: c-MYC inhibition impairs hypoxia response in glioblastoma multiforme
Source: Oncotarget. 2016 Apr 22;7(22):33257–71. doi: 10.18632/oncotarget.8921 (PMC5078092; doi:10.18632/oncotarget.8921)
Supplement: Supplementary file 1 [file oncotarget-07-33257-s001.pdf]

SUPPLEMENTARY METHODS

Cell transfection and infection

U373GFP and U373FO cells were obtained by infection with lentiviral particles encoding Flag tagged Omomyc and Green Fluorescent protein (GFP) as described for U87MG in Experimental Procedures.

A pcDNA3.1 plasmid encoding HIF1A-mut was kindly provided by Dr. W.G. Kealin. In this mutant proline 402 and proline 564 were substituted by alanine. Prolines 402 and 564, when hydroxylated, generate a recognition motif for the VHL ubiquitin ligase, therefore HIFA-mut is not constitutively ubiquinated in normoxia and has an increased half-life. The sequence encoding HIF1A-mut was sub-cloned in pLPCX retroviral vector (Clontech, USA). 293T cells were transfected with the pLPCX-HIF1A-mut plasmid together with pGAG and pVSVG plasmids using Lipofectamine reagent (Invitrogen). Forty-eight hours post-transfection retroviral particles were collected and used to infect U87FO cells. U87FO cells constitutively expressing HIF1A-mut protein were obtained upon selection with puromycin (Sigma).

Sequences encoding short hairpin RNAs targeting c-MYC (siMYC 5′-acgacgagaccttcatcaa-3′) and or a control short hairpin (siR5 5′-gggatatccctctagatta-3′) were cloned in a pRETROper vector bearing the puromycin resistance gene. Hela cells were transiently transfected with the pRETROper plasmids using Lipofectamine reagent (Invitrogen) and 48h post-transfection selected for four additional days with 1μg/ml puromycin.

List of oligonucleotides used in real-time PCR

Gene symbol	Sequence
CA9 For	5′-tgcctatgagcagttgctgt-3′
CA9 Rev	5′- tgggacctgagtctctgagc-3′
PGK1 For	5′- tggatcttgtctgcaactttagc-3′
PGK1 Rev	5′- tgcaaaggccttggagag-3′
DDIT4 For	5′- ctggacagcagcaacagtg-3′
DDIT4 Rev	5′- tcactgagcagctcgaagtc-3′
NDRG1 For	5′- cctacatcctaactcgatttgctc-3′
NDRG1 Rev	5′- gggttcacgttgataaggaca-3′
TBP For	5′- tgcccgaaacgccgaatataatc-3′
TBP Rev	5′- tggttcgtggctctcttatcctc-3′

SUPPLEMENTARY FIGUERS AND TABLES

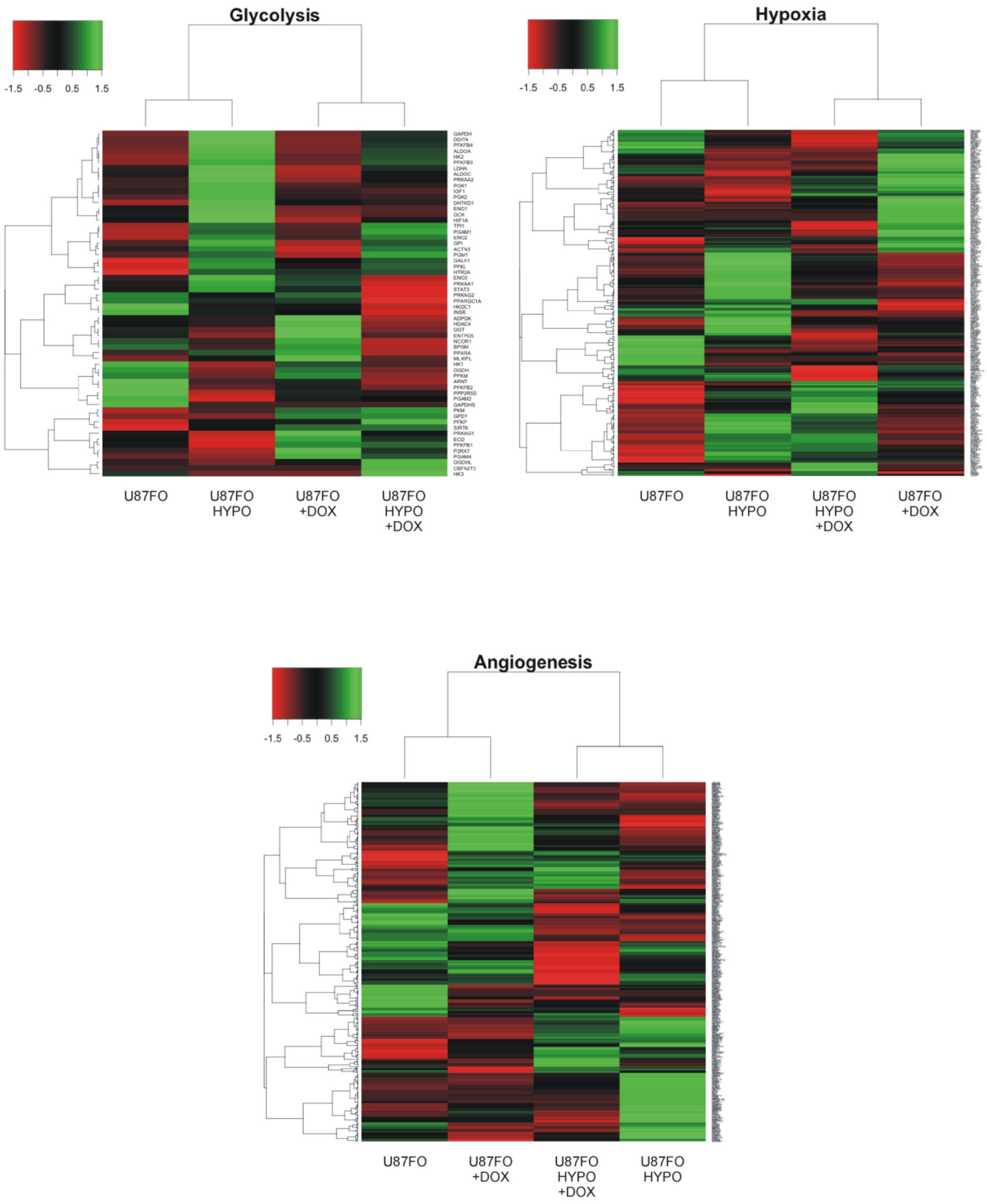

Supplementary Figure S1: RNA-seq analysis of selected sets of hypoxia regulated genes. Expression heatmaps for human genes associated to response to hypoxia (GO:0001666), angiogenesis (GO:0001525) and glycolysis (GO:0006096) gene ontology terms. Gene sets were retrieved from AmiGO. Expression values are shown as per-row FPKM Z-scores using a color scale from red (low Z-score) to green (high Z-score).

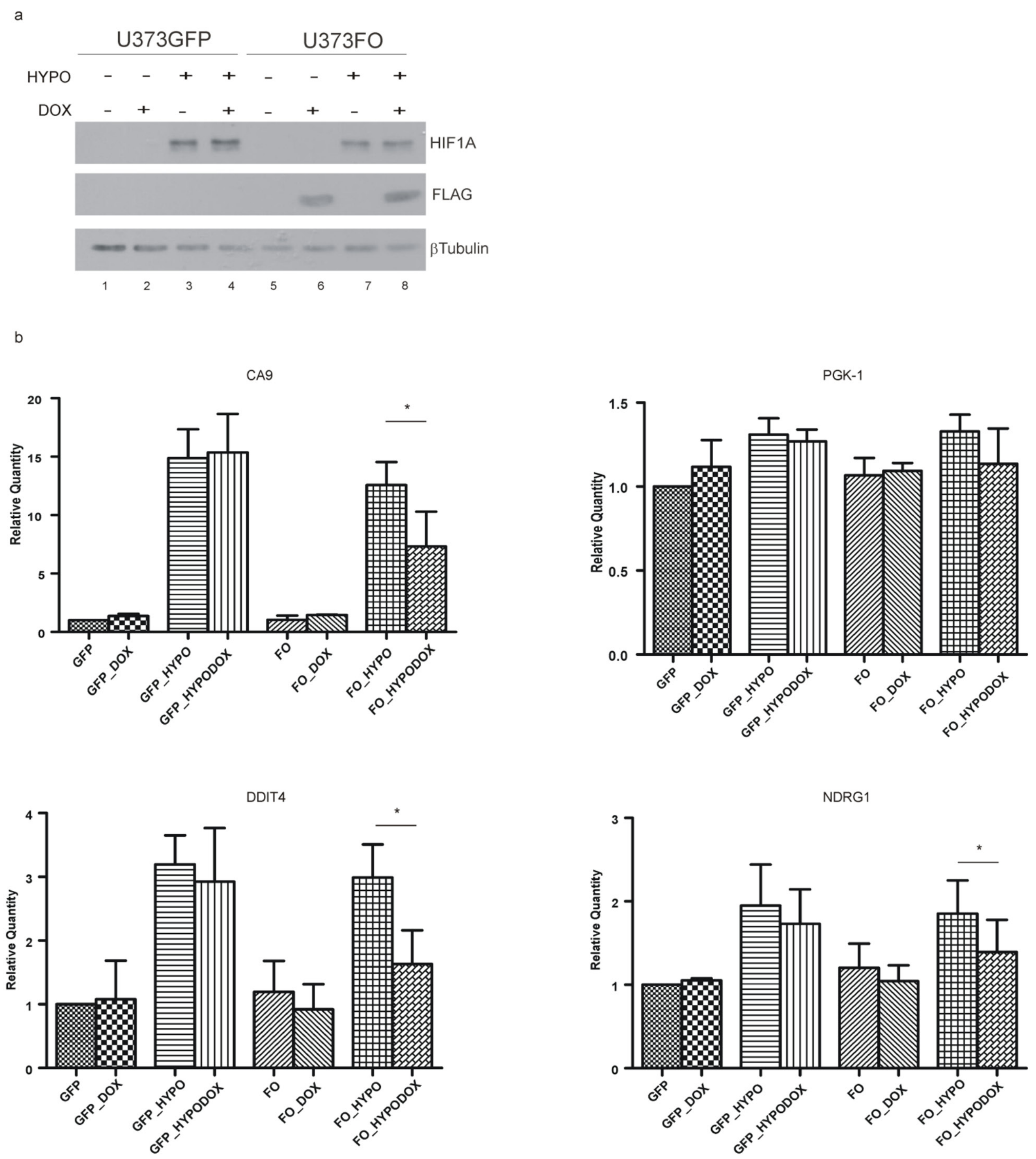

Supplementary Figure S2: c-MYC regulates HIF1A transcription activity in U373 cells. a. Western blot analysis on U373GFP and U373FO cells pretreated with DOX and then exposed to hypoxia. DOX treatment caused Omomyc expression (Lane 6 and 8). Hypoxia-induced HIF1A protein stabilization was not altered by Omomyc expression (Lane 3, 4, 7 and 8). **b.** Quantitative Real Time RT-PCR on HIF1A target genes, in U373GFP cells and U373FO cells. CA9, PGK-1, DDIT4 and NDRG1 are shown. Relative Quantity was calculated relative to TBP and is given relative to U87GFP. * p value < 0.05.

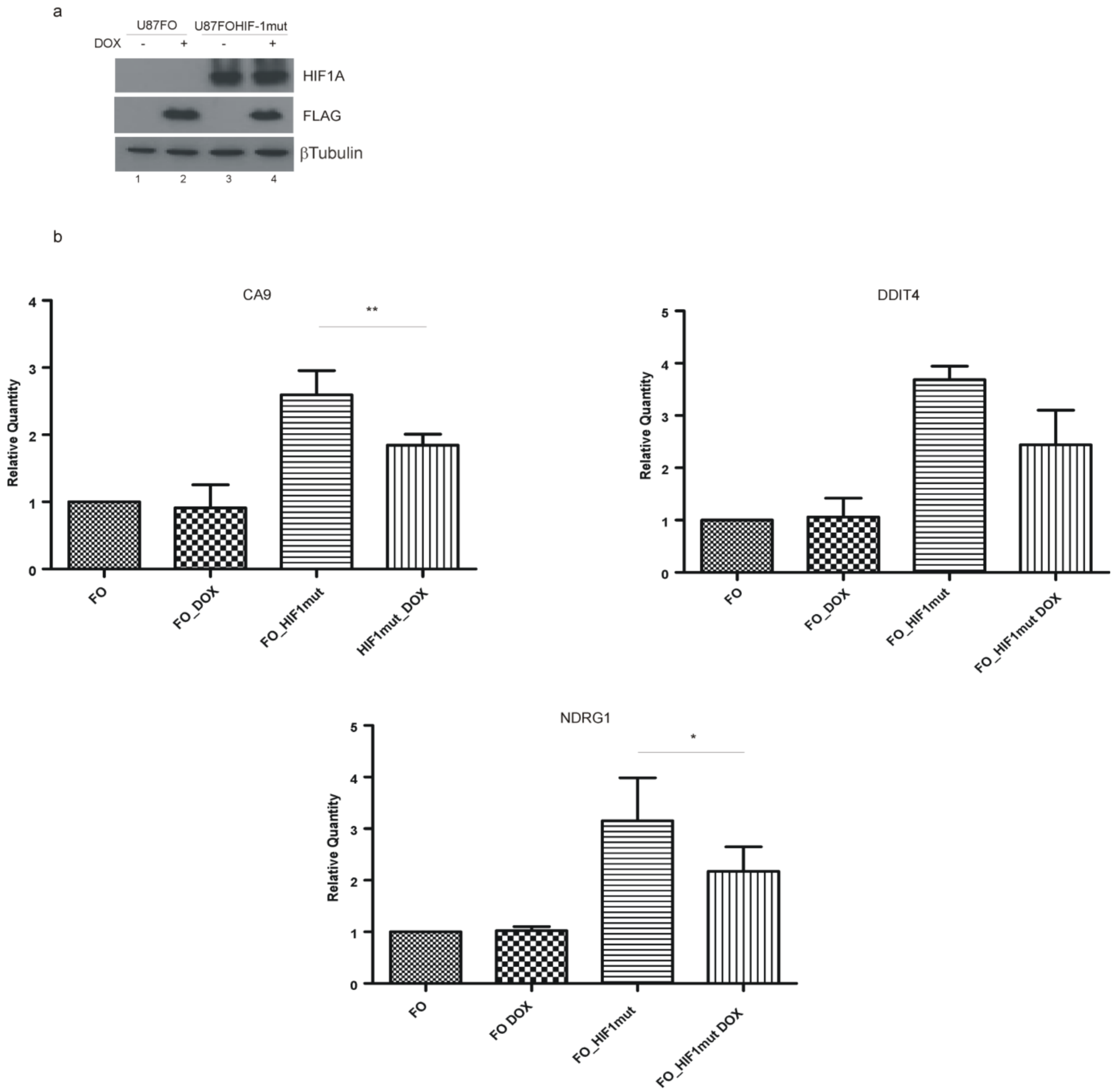

Supplementary Figure S3: c-MYC inhibition impairs HIF1A dependent gene transcription in the absence of hypoxia. **a.** Western blot analysis of U87FO cells expressing a normoxic stable mutant HIF1A (HIF1Amut), lanes 3 and 4. DOX- induced Omomyc expression was also detected (Lanes 2 and 4). **b.** Quantitative Real Time PCR performed in U87FO cells and U87FO-HIF1Amut cells are shown. HIF1A target genes were induced by HIF1A-mut expression and this induction was blunted in Omomyc expressing cells. Relative Quantity was calculated relative to TBP. *p<0.05 and **p<0.01.

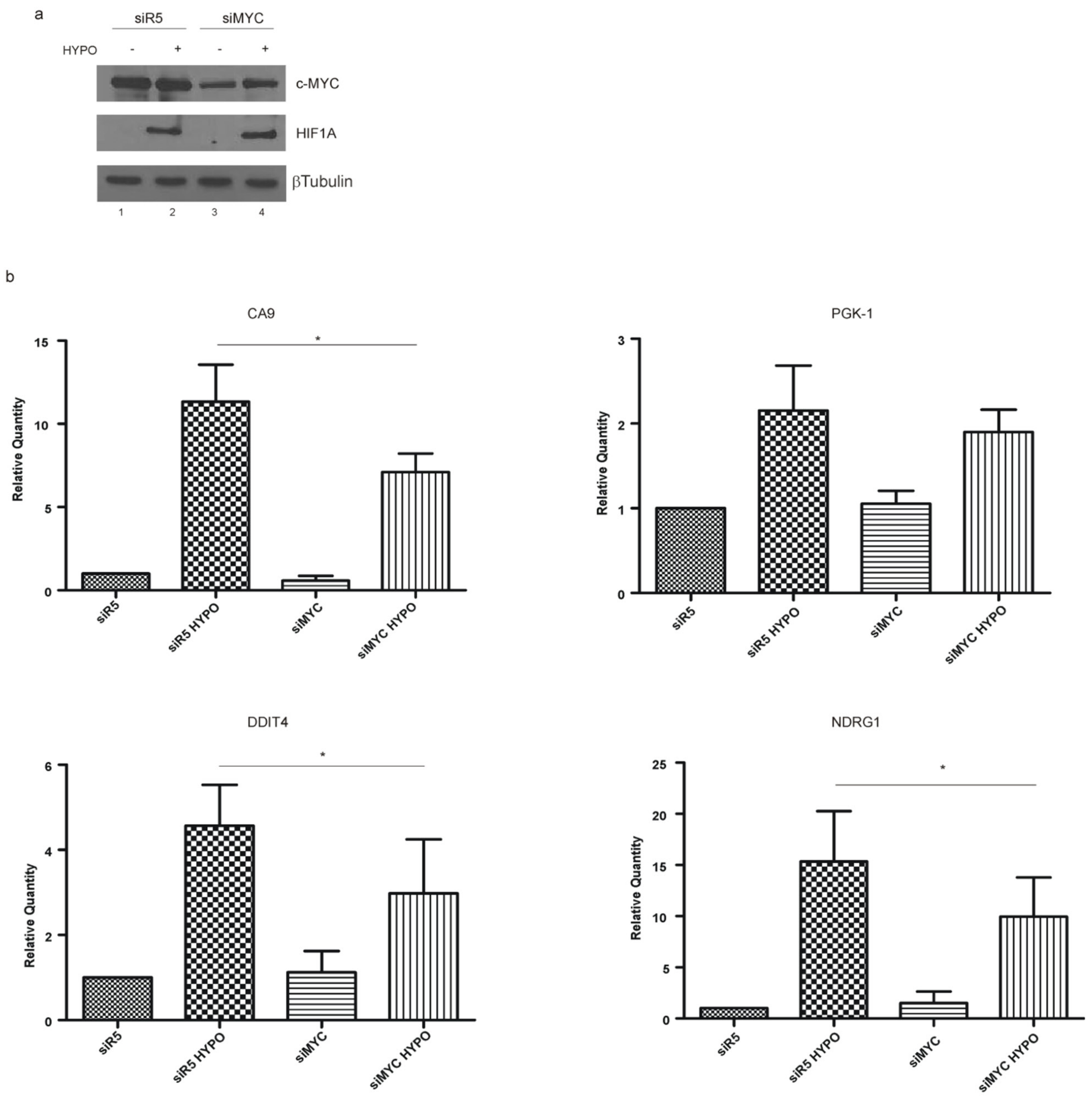

Supplementary Figure S4: Hypoxic induction of Omo-down genes expression in c-MYC RNA interfered HeLa cells. The analysis was performed in Hela cells because they can be transfected with high efficiency. **a.** Western blot analysis on HeLa cells transfected with control shRNA, siR5, (Lanes 1 and 2) and with siMYC (Lanes 3 and 4). Cells were kept in normoxia (Lanes 1 and 3) or exposed to hypoxia for 5h (Lanes 2 and 4). **b.** Quantitative Real Time PCR on HeLa cells transfected with siR5 and with siMYC. Relative Quantity was calculated relative to TBP. *p<0.05.

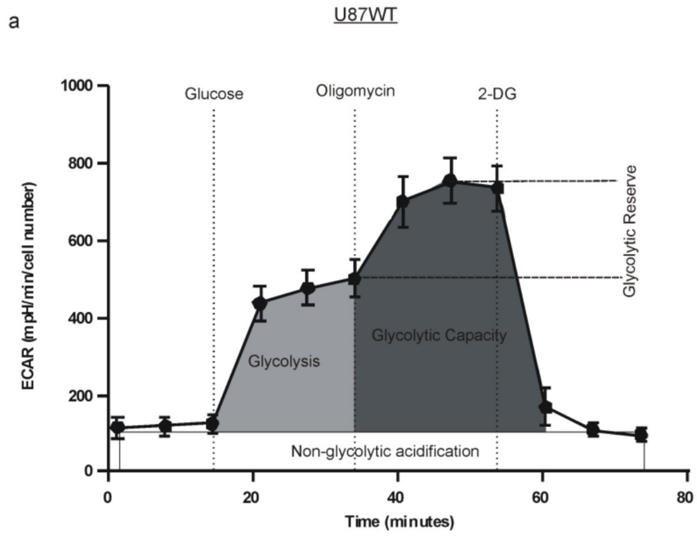

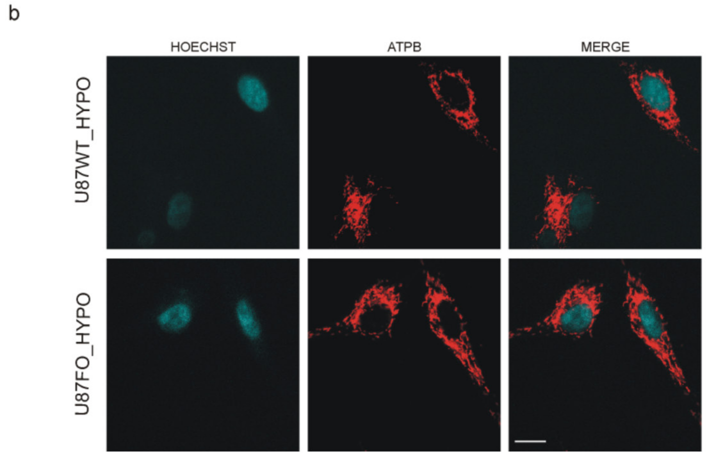

Supplementary Figure S5: Metabolic characterization of U87MG expressing or not Omomyc. a. Extracellular acidification rate (ECAR) of U87MG cells under standard conditions. Areas in different grey scale describe the metabolic analysis that can be extrapolated based on ECAR values after glucose, oligomycin and 2-DG injections. Data show mean ± SD. **b.** Immunofluorescence analysis of ATPB mitochondrial protein on U87WT cells and U87FO cells exposed to hypoxic conditions for 16h. Scale bar, 10µm.

Supplementary Table S1: Analysis of randomly generated gene sets

	OMO-DOWN			OMO-UP		
	**Avg exp**	**Random>Avg**	**Random<Avg**	**Avg exp**	**Random>Avg**	**Random<Avg**
**Classical**	**-0.0176**	6703	3297	**0.0524**	1443	8557
**Mesenchymal**	**0.0768**	2277	7723	**0.2061**	188	9812
**Neural**	**-0.01391**	9866	134	**-0.0344**	3910	6090
**Proneural**	**0.0188**	1728	8272	**-0.277**	9999	1

Comparison of the mean expression of Omo-down and Omo-up gene sets and the expression of 10000 randomly generated sets of the same size.

Supplementary Table S2: DCA gene set enrichment in hypoxic U87FO cells

Gene set	NES	FDR q value
**Genes by DCA treatment**	-1.1	0.17

GSEA of the gene set defined by DCA response in hypoxic U87FO cells versus Omomyc-expressing hypoxic U87FO cells.
